# Injectable peptide-glycosaminoglycan hydrogels for soft tissue repair: *in vitro* assessment for nucleus augmentation†

**DOI:** 10.1039/d4ma00613e

**Published:** 2024-10-10

**Authors:** James P. Warren, Ruth H. Coe, Matthew P. Culbert, Andrew R. Dixon, Danielle E. Miles, Marlène Mengoni, Paul A. Beales, Ruth K. Wilcox

**Affiliations:** a Institute of Medical and Biological Engineering, School of Mechanical Engineering UK J.P.Warren@leeds.ac.uk; b School of Chemistry, University of Leeds Leeds LS2 9JT UK

## Abstract

We report the development of peptide-glycosaminoglycan hydrogels as injectable biomaterials for load-bearing soft tissue repair. The hydrogels are injectable as a liquid for clinical delivery, rapidly form a gel *in situ*, and mimic the osmotic swelling behaviour of natural tissue. We used a new *in vitro* model to demonstrate their application as a nucleus augmentation material for the treatment of intervertebral disc degeneration. Our study compared a complex lab gel preparation method to a simple clinical benchtop process. We showed pH differences did not significantly affect gel formation, and temperature variations had no impact on gel performance. Rheological results demonstrated consistency after benchtop mixing or needle injection. In our *in vitro* disc degeneration model, we established that peptide augmentation could restore the native biomechanical properties. This suggests the feasibility of minimally invasive peptide-GAG gel delivery, maintaining consistent properties across temperature and needle sizes while restoring disc height and stiffness *in vitro*.

## Abstract 2

We developed injectable peptide-glycosaminoglycan hydrogels that mimic natural tissue swelling and rapidly gel *in situ*, that were able to restore the biomechanical properties in an *in vitro* bovine disc degeneration model.

## Graphical abstract

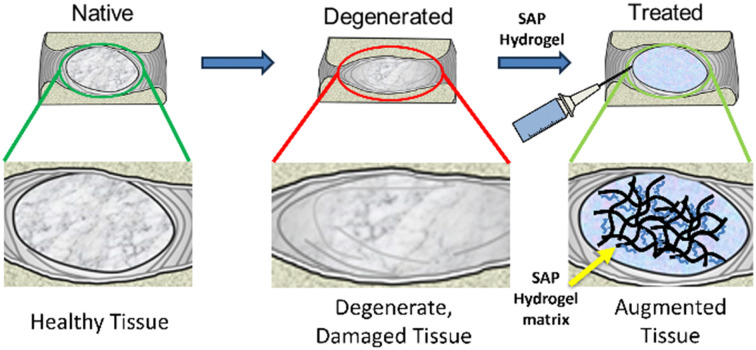

## Introduction

Injectable biomaterials have shown great promise in minimally invasive treatments as carriers for drugs or cells.^1,2^ While they have potential for use directly as devices for tissue repair, there are challenges in meeting the mechanical requirements for load-bearing applications, particularly in musculoskeletal tissues.

In soft tissues such as articular cartilage and the intervertebral disc, the fluid component plays a critical role in governing the mechanical behaviour. These tissues contain high concentrations of proteoglycan macromolecules with negatively charged glycosaminoglycan (GAG) side chains that draw water into the tissue and provide a swelling pressure.^3^ Degeneration and disease can reduce the size and quality of the proteoglycan aggregates, resulting in a loss of swelling pressure and a cascade of further biomechanical, chemical and biological changes.^4^

Any treatment in which the degenerated tissue is replaced or augmented by a biomaterial must therefore be able to mimic the fluid as well as the solid components of the structure.

In the case of the intervertebral discs, there is an association between these degenerative changes and back pain, especially in the lower spine.^5,6^ Back pain is ranked as the leading cause of years lived with disability,^7^ and the total costs associated with the condition are estimated to be over US$ 100 billion per year in the US alone.^8^ Despite the scale of the problem, there are limited clinical approaches to prevent or treat progressive degeneration of the discs.

**Fig. 1:**
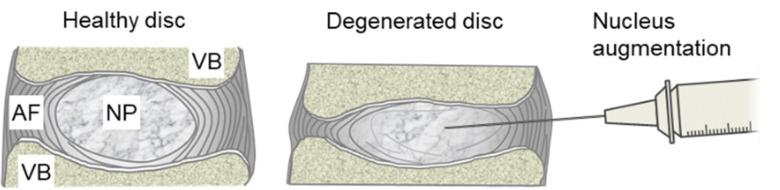
The intervertebral discs are located between vertebral bodies (VBs). The healthy intervertebral disc comprises a GAG-rich nucleus pulposus (NP) surrounded by the annulus fibrosus (AF). In the degenerated disc, there is a reduction in GAGs and the disc loses height. The concept of nucleus augmentation is to increase disc height and restore functionality through the minimally invasive injection of a hydrogel into the NP region of the degenerated disc.

The intervertebral discs are the soft tissues between the vertebrae that allow their articulation. They comprise an outer annulus fibrosus, a layered structure of collagen fibres aligned in alternating orientations, and an inner gel-like nucleus pulposus (Fig. 1). Degenerative changes cause the nucleus to lose GAGs and result in a loss in the overall disc height.

End-stage surgical treatment most commonly involves the fusion of the two adjacent vertebrae, but this may lead to accelerated degeneration at adjacent levels and has relatively poor clinical outcomes.^9^ A number of regenerative therapies that aim to restore disc homeostasis have been investigated, but these are challenged by the avascular nature of the tissue, which limits nutrient supply.^10^

Some groups have attempted to promote disc regeneration through the injection of various cell types encapsulated within synthetic biomaterials, including chitosan/gelatin crosslinked,^11^ modified poly-(*N*-isopropylacrylamide),^12,13^ dextran/chitosan/teleostean,^14,15^ synthetic genipin-crosslinked fibrin hydrogels and another self-assembling peptide variant hydrogel.^16,17^ These gels have been shown to support accelerated cell growth but currently do not match the mechanical properties of human intervertebral discs.

We have previously shown that a class of self-assembling peptide hydrogels can be designed to mimic the natural properties of hydrated soft tissues when combined with GAGs.^18,19^ Importantly, the presence of GAGs not only mimics the natural tissue's ability to imbibe water, but also enhances the thermodynamic stability and gelation kinetics of the peptide.^20^

**Fig. 2:**
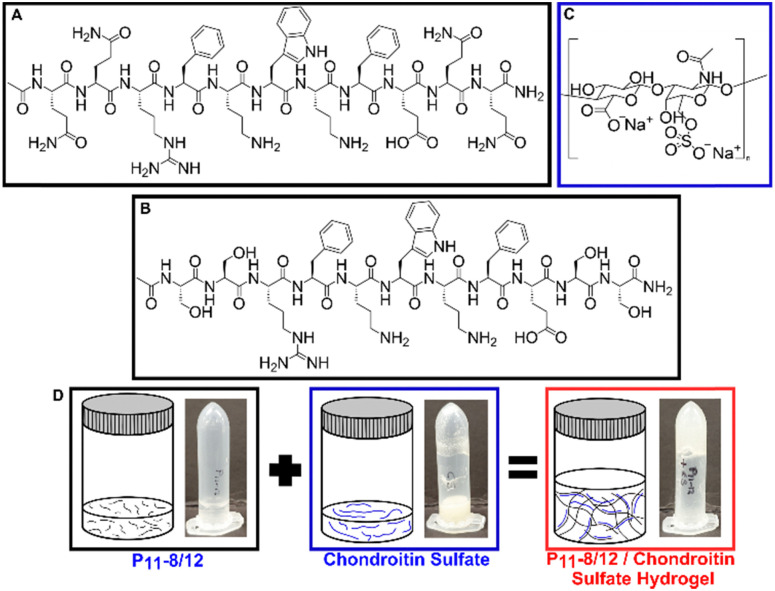
Structural representation of the peptides ((A) P_11_-8 and (B) P_11_-12) and (C) chondroitin sulfate. (D) Graphical representations and visual photographs of each component and upon mixing.

Through using differing peptide : GAG ratios, the range of GAG concentrations naturally found in human intervertebral discs can be replicated. A structural representation of the peptides is shown in Fig. 2. Fig. 2A and B show the structures of P_11_-8 and P_11_-12 respectively, while Fig. 2C shows the structure of chondroitin sulfate. Fig. 2D illustrates the physical state of the peptide and GAG molecules prior to mixing. Upon mixing, the two components interact which results in the self-assembled hydrogel.

**Fig. 3:**
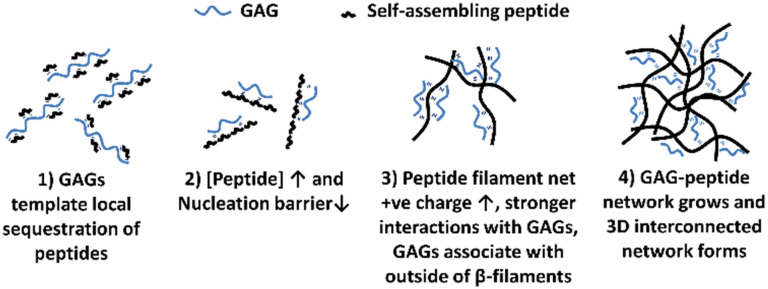
Graphical representation of the hypothesised mechanisms of interactions between peptide and GAG molecules upon mixing. (1) GAGs template initial peptide association and aggregation. (2) When [peptide] > critical concentration (c*), nucleation barrier lowers, catalysing self-assembly. (3) Increased net charge allows stronger interactions to occur between growing peptide chains and GAGs. (4) Peptide filaments interact and crosslink to form a 3D gel network.

The mechanism of interaction between the peptide and GAG molecules is currently poorly understood, however we propose a hypothesis behind the mechanism which is illustrated in Fig. 3. This hypothesis is based on previously reported experimental observations when varying concentrations of different charged peptides, both negative and positive, and GAGs were combined.^18–21^ These previous results indicated that in low peptide concentration regimes, below a critical concentration (c*), the inclusion of GAGs reduced the peptide concentration needed for spontaneous self-assembly into structures *via* an anti-parallel beta-sheet intermediate, as shown by nuclear magnetic resonance (NMR) and Fourier transformed infra-red (FT-IR) spectroscopy.^20,21^ Transmission electron microscopy (TEM) imaging confirmed nanoscale tapes and ribbons in these low concentration samples with GAGs. At higher peptide concentrations, TEM imaging revealed that GAGs promoted the formation of denser, more extensive fibrillar networks compared to peptide-only samples.^18,19^ From these results, we hypothesise that: (1) the larger GAGs act as a template for local sequestration and concentration of peptides through polyelectrolyte complexation and hydrogen bonding. However this interaction with individual peptides (net charge +2*e*) is weak and reversible, allowing the peptides to explore other local favourable interactions within liquid-like condensates. (2) This sequestration of peptides by the GAGs increases their local concentration and lowers the nucleation barrier thereby catalysing assembly of peptide β-fibrils. (3) The self-assembled peptide filaments then carry a much larger net positive charge that increases the strength of polyelectrolyte complexation with the polyanionic GAGs, such that the GAGs then decorate the outside of the peptide β-filaments. (4) The growing GAG-decorated peptide filaments then interact and crosslink the 3D gel network, where the GAGs are long enough to bind to multiple peptide filaments, enabling bundling and crosslinking interactions at points of connection in the network.

We have demonstrated that the hydrogels can be formulated to match the mechanical properties of the natural nucleus pulposus and have potential as a treatment for intervertebral disc degeneration (Fig. 1). Here, we report the development of peptide-GAG hydrogels that meet the concurrent requirements of being injectable as liquids for clinical delivery, rapidly (<10 s) and reliably forming a gel *in situ*, and mimicking the swelling behaviour of natural tissue.^22^ We specifically examine the performance of the gels as a minimally invasive therapy for intervertebral disc degeneration.

In a static *in vitro* model, we have demonstrated that denucleated discs augmented with the peptide-GAG hybrid hydrogels exhibit properties similar to the native tissue.^20,23^

However, the evaluation of the biomechanical performance of biomaterials for nucleus pulposus augmentation or replacement is hampered by the lack of standard laboratory testing methodologies.^24^*In vitro* models employing cadaveric or large animal intervertebral disc specimens have been used to mimic the natural physiological environment.^25–30^ These models have been tested under cyclic loading, using either biochemical or mechanical approaches to simulate disc degeneration.^24^ However, the direct effects of an intervention are often masked by the large variations in mechanical behaviour seen across specimens, due to anatomical variances and changes in specimen hydration.^23^ Here, we propose a new accelerated testing approach which enables longitudinal comparisons of the same specimen in different states, while minimising the test durations.

Finally, we report on the use of this methodology to assess the biomechanical performance of the peptide-GAG hydrogels and their ability to restore artificially degenerated tissue to the healthy state.

## Experimental sections

### Hydrogel materials

The peptides were custom synthesised (CS Bio, USA). Peptide quality control was undertaken by the synthesis company. The peptide content reflects non-peptide molecules present in the dry peptide mass; these were mainly residual amounts of water and trifluoroacetic acid (TFA) counterions.

The GAG used throughout this study was a chondroitin sulfate (CS) sodium salt from shark cartilage (*M*_w_ ∼ 58 kDa) (Sigma Aldrich, UK).

### Hydrogel preparation methods

To examine the effects of preparation, two methods were compared.

The established laboratory protocol^12^ incorporated pH-switching and heat monomerisation. The peptide powder and NaCl solution (130 mM) was vortexed and sonicated, followed by pH adjustment to 7.4 ± 0.05, heated to 80 °C and further vortexed. The CS powder and NaCl solution (130 mM) was vortexed until dissolved. The two solutions were pipetted together and further vortexed until homogenous.

In the vortex only method, both the peptide-NaCl and CS-NaCl solutions were vortexed until clear and the peptide solution was then added to the GAG solution with further vortexing until homogenous.

For the needle delivery and rheology studies, the peptide and GAG solutions were separately prepared. Both peptide and GAG solutions were vortexed for 30 seconds, sonicated to remove air bubbles for 1–2 min, then drawn into 1 mL syringes and the relevant needle attached.

### Fourier transformed infra-red (FT-IR) spectroscopy

For FT-IR analysis, samples were made up using D_2_O (Sigma-Aldrich, UK) instead of H_2_O, to lower the band (1630 cm^−1^) associated with bending outside of the amide I’ region.

Samples were placed between two CaF_2_ windows (thickness = 3 mm each) with a copper spacer in between the windows (thickness = 0.25 mm) and their transmission spectra acquired four days after preparation with a Thermo Scientific Nicolet 6700 FTIR spectrometer. Spectra were averages of 32 scans recorded at room temperature. Blank solvent (130 mM NaCl in D_2_O) spectra were subtracted from the sample trace, the baseline corrected and the spectra smoothed. Processed spectra were band fitted in the amide I’ region (1720–1580 cm^−1^) using the peak resolve routine in OMNIC7.3 SP1 (Thermo Electron Corporation), providing information on the number and positions of individual component bands. The peak positions corresponding to secondary structures used to determine β-sheet content are presented in Table 1.

**Table 1:** Peak positions used to determine β-sheet content

Amide I’ band (cm^−1^)	Secondary structure assignment
1613–1630	β-Sheet
1642–1649	Unordered
1649–1655	α-Helix
1658–1674	Turn
1682–1690	Anti-parallel β-sheet
1694–1697	Turn

Note that in the purification of peptides, trifluoracetic acid, TFA is used, which leads to it being present in the peptide material as a counter ion bound to the positively charged residues. TFA has a FTIR band located at 1673 cm^−1^ and peptides with greater number of arginine and ornithine residues will contain more TFA and therefore a large TFA peak in the FTIR spectra.

### Rheology

Peptide-GAG samples were made by injecting the two individual solutions into a 2 mL Eppendorf using a syringe driver. Samples were made 24 hours prior to testing and maintained at room temperature before being loaded onto the rheometer using a custom increased diameter 1 mL Eppendorf pipette tip.

Rheology measurements used a Malvern Kinexus Pro rheometer with a cone-plate geometry (cone angle: 1°, diameter: 50 mm, gap: 0.03 mm). All tests were performed at 25 °C, utilizing a solvent trap. The atmosphere within was kept saturated to minimize evaporation of the peptide samples. The cone was lowered into position and samples incubated for 15 min. To ensure measurements were made in the linear viscoelastic regime, amplitude sweeps were performed in a shear strain controlled mode from 0.01–100% at 1 Hz and 20 Hz. The dynamic moduli of the hydrogels were measured as a frequency function with the sweeps carried out between 1 and 20 Hz.

The shear moduli for the gels produced using different needle configurations was compared using a two-way ANOVA tests with Tukey *post hoc* analysis (*p* ≤ 0.05). Statistical analysis was carried out using Origin 2019 software (OriginLab Corporation, USA).

### Needle delivery studies

A few drops of food colouring were added to the peptide (blue) and CS (yellow) solutions prior to vortexing. Needles were inserted at different measured orientations into an Eppendorf and the two solutions were injected simultaneously. Photographs of the resulting gel were taken to examine the consistency of mixing.

Further tests were undertaken using a bovine tail bone-disc-bone unit. First, a trans-endplate nucleotomy was undertaken, in which a 10 mm diameter central region of the nucleus was removed by drilling through the superior vertebra and endplate, avoiding damage to the inferior endplate.^22^ A camera was mounted above the specimen allowing visualisation of the nucleus void. The peptide-GAG hydrogel was then injected into the void through two parallel 25 G needles using a syringe driver, and the process filmed to observe the gelation.

### Biomechanical testing

#### Bovine disc preparation

Bovine tails were harvested from calves aged less than 30 months at a local abattoir and frozen (−80 °C) prior to experimentation. The variation between specimens was minimised by using only the most cranial four levels. The tails were cleaned and imaged under microCT, using a bespoke rig, to identify the positioning of the sections to ensure consistent 15 mm lengths of bone were retained on either side of the disc.^23^ The bone-disc-bone units were then excised *via* transverse cuts through the vertebrae. Specimens were prepared by cleaning the blood and marrow using a water pik followed by a 24 hours soak in sodium citrate solution (20.5 mM, pH 7.4) under agitation at 4 °C. Prepared specimens were frozen until testing at −80 °C.

#### Mechanical testing

The following steps were then undertaken on the specimens in their native state and again after artificial degeneration and after treatment. First, to allow the specimens to reach osmotic equilibrium prior to mechanical testing, they were held under a ∼40 N load in a PBS bath at 37 °C for 24 hours using a custom rig. Cyclic compression tests were then undertaken on a dynamic materials testing machine (ElectroPuls E10000, Instron, UK) under load control between 356 and 744 N at 1 Hz for 100 cycles. The upper and lower limits were selected to produce intradiscal pressures representative of high (carrying 20 kg of weight) and low (unsupported sitting) loading activities.^31–33^ During testing, specimens were immersed in a PBS bath at 37 °C and porous fixtures were used to allow fluid flow through the endplates.

#### Degenerative model

**Fig. 4:**
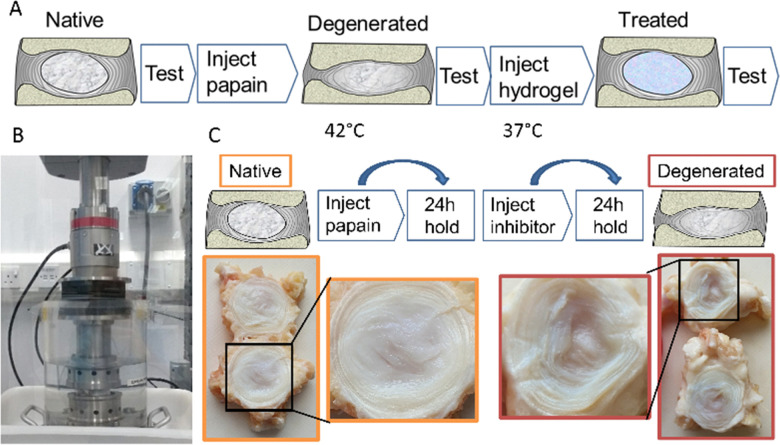
(A) Schematic of the *in vitro* longitudinal testing protocol. (B) Specimens were cyclically loaded in a heated fluid bath under axial compression in a materials testing machine. (C) The degeneration model involved two steps: enzyme (papain) injection and inhibitor injection. The degenerated nucleus is highlighted, post-enzymatic digestion.

The specimens were artificially degenerated by injecting a concentrated papain solution (0.3 ml, 1.6 kU ml^−1^) to non-selectively break down collagen and proteoglycan structures within the nucleus pulposus. The papain solution was injected through a 30 G needle into the nucleus of the disc and the specimens were then held under a ∼40 N load at 42 °C for 24 hours, followed by the injection of an ebselen inhibitor solution (0.3 ml, 2.13 mM) to stop further enzymatic activity. This process is illustrated in Fig. 4.

#### Hydrogel injection

Peptide-GAG hydrogels were injected as a 2-part solution through two 25 G 100 mm long needles aligned in parallel, representing the minimally invasive clinical delivery with needles of sufficient length to reach the centre of the nucleus pulposus from a superio-lateral approach. Tests were undertaken on both P_11_-8 and P_11_-12 (both at 1 : 20 peptide : GAG ratio); a radio-opaque agent (Ultravist® 300, Bayer PLC, Reading, UK) was mixed with NaCl solution (130 mM) and carboxyfluorescein NaCl solution (2.7 mM) in a ratio of 1 : 2 : 1. This solution was then mixed with the hydrogel prior to injection to enable visualisation using microCT.

#### Imaging

Specimens were imaged during the testing sequence using micro-CT (uCT100, Scanco Medical, Switzerland).

#### Data analysis

Cyclic data was post-processed to extract specimen stiffness over the loading portion of each cycle, and the average stiffness over the last 10 cycles (*i.e.* at closest to steady-state) was used for analysis. The specimen height was also measured at each stage of testing. A two-factors repeated measure ANOVA was used to compare the stiffness values or the specimen height across the three testing stages and the two types of peptide used. Statistical analysis was performed using R.4.1.1 (R Project for Statistical Computing, R Foundation, https://www.r-project.org) after testing for data normality with a Shapiro-Wilk test and model sphericity with a Mauchly test.

## Results and discussion

We compared the gels prepared using our established laboratory protocol incorporating pH switching and heat monomerisation,^20^ and those prepared with a simpler vortex-only benchtop process more suitable for clinical use.

**Fig. 5:**
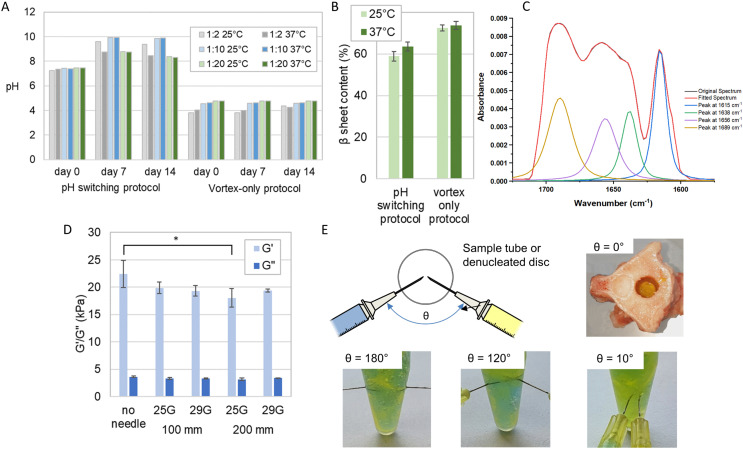
Evaluation of the clinical mixing and delivery of the P_11_-12-GAG hydrogel prepared using vortex only protocol. (A) The pH measured over a period of 14 days of hydrogels prepared at different temperatures using the pH-switching method reported previously^11^ and a simplified vortex-only process. (B) The corresponding β sheet content of the 1 : 20 ratio samples (mean ± S.D., *n* = 3), measured from Fourier transform infra-red spectra. (C) FTIR spectrum of P_11_-12-GAG hydrogel, highlighting amide I region between 1650–1750 cm^−1^ delivered through different needle diameters and lengths (mean ± S.D., *n* = 3), measured with a cone-on-plate geometry rheometer * = significant (*p* < =0.05). (D) The elastic (*G*′) and viscous (*G*′′) components of shear modulus of 1 : 20 ratio. (E) The evaluation of different orientations of needle delivery using dye to differentiate the GAG (yellow) and peptide (blue) components and instantaneous gel formation after injection into a denucleated disc.

We observed distinct pH variations (Fig. 5A) in the pH switching method, with all samples showing an increase in pH at both 25 °C and 37 °C. This increase extended from a range of pH 7.2–7.4 at day 0 to pH 8.7–9.9 at day 7, after which it stabilized until day 14. Notably, at day 7 and day 14 for both the 25 °C and 37 °C samples with the lowest GAG concentration (1 : 2), there was a pH decrease from pH 9.6 (25 °C) to pH 8.7 (37 °C). Conversely, there was no measurable pH change observed from day 0 to day 14 for samples with mid and high GAG concentrations (1 : 10 and 1 : 20) at both temperatures in the vortex-only protocol. At the lowest GAG concentration (1 : 2), the samples at both temperatures exhibited a pH increase from pH 3.8 to 4.3 (25 °C) and pH 4.0 to 4.2 (37 °C) between day 7 and day 14. However, despite these pH variations, no observable alterations in the formation of a self-supporting gel were noted between the two preparation methods. The pH variation from day 0 to day 7 indicated that gels made with the pH switching protocol did not reach a steady-state until at least day 7, unlike those made with the vortex-only protocol, which were stable earlier. This suggests that vortex-only gels organise their molecular structuring more quickly without significant further ripening on longer time scales, an important factor for the clinical potential of injectable gels.

Crucially, the analysis revealed that there were no statistically significant distinctions in β-sheet content (%) between samples prepared at 25 °C and 37 °C (Fig. 5B and C). This lack of disparity was consistent across both preparation methods. In the pH switch method, the β-sheet content (%) for samples at 25 °C and 37 °C was 58.9 ± 2.3 and 63.5 ± 3.2, respectively. Meanwhile, in the vortex-only method, the β-sheet content (%) for samples at 25 °C and 37 °C stood at 72.5 ± 1.4 and 73.7 ± 1.9, respectively. Furthermore, it is noteworthy that these differences in β-sheet content were smaller with the vortex-only protocol, particularly at higher GAG concentrations (Fig. 5A and B). These results indicate that variances in operating room temperatures and handling would not affect the performance of the gel.

Previous studies have shown that the mode of agitation during peptide gel transition can dramatically influence the mechanical properties of the resulting gel.^19,34^ We hypothesised that the shear forces applied to the monomer and GAG solution during injection could also affect peptide self-assembly the subsequent gelation.

We examined the rheological properties of the hydrogels prepared through a standard benchtop vortexing method and following injection down fine-gauge needles, at a flow rate of 0.22 ml min^−1^, with no external agitation. In all cases, self-supporting gels were found to form. The flow rate was kept constant through using an automated syringe driver.

Variations in the rheological properties when comparing the benchtop prepared hydrogels to those formed after injection through various needles are shown in Fig. 5D. The sole significant difference (*p* < 0.05) emerged between the absence of a needle and the use of a 25 G needle with a 200 mm length. In contrast, no other significant variations were observed in the rheological properties concerning the needle's length and gauge when preparing the gels.

The viscosity of the gels, prepared with or without a needle of any gauge or length, ranging from 18 kPa–22 kPa were comparable to that of the natural nucleus pulposus, which is approximately 19 kPa^34–37^ (Fig. 5D).

We explored the effect of injection order and needle orientation on gel uniformity (Fig. 5E). Sequential injection, with the CS solution preceding the peptide solution, resulted in a layered structure at the interface. The peptide layer self-assembled but did not blend with the CS solution beyond the interface, creating distinct yellow and blue layers with a green band at the interface, especially evident in Eppendorf samples. Conversely, reversing the injection order improved mixing and gelation, yielding a larger volume of the green-coloured gel, although some regional distinctions persisted. Simultaneous injection led to a completely homogeneous green gel. We also assessed the effect of needle angle. At 180°, heterogeneous mixing produced yellow and blue regions within the green gel. At 120°, separation akin to sequential injection occurred, while at 10°, homogeneous mixing created a uniform green gel. These findings illuminate how injection order and needle angle influence gel uniformity and homogeneity.

Finally, we used a dual needle injection system to deliver the gels simultaneously down 100 mm 25 G needles. We showed consistent mixing of the gels in an Eppendorf. Furthermore, we saw instantaneous gel formation when injected into a denucleated intervertebral disc that had been sectioned transversely to allow viewing (Fig. 5E). This dual delivery system maximises consistency by ensuring co-location of the needle tips and equal delivery rates of the two components using a dual syringe. It also has the greatest clinical applicability given it would require a single insertion procedure.

For the biomechanical testing, the degeneration model utilised papain as a broad protease to non-selectively break down proteins, mainly collagen, within the nucleus and inner annulus. The enzymatic inhibitor deactivated the papain to stop the enzymatic digestion at a known time point. The route allowed accurate control of the digestion process, minimising variation between specimens.

As illustrated photographically in Fig. 4, this process was found to cause degeneration of the nucleus, with a characteristic fluid-filled void present where the protein structure had previously been.

**Fig. 6:**
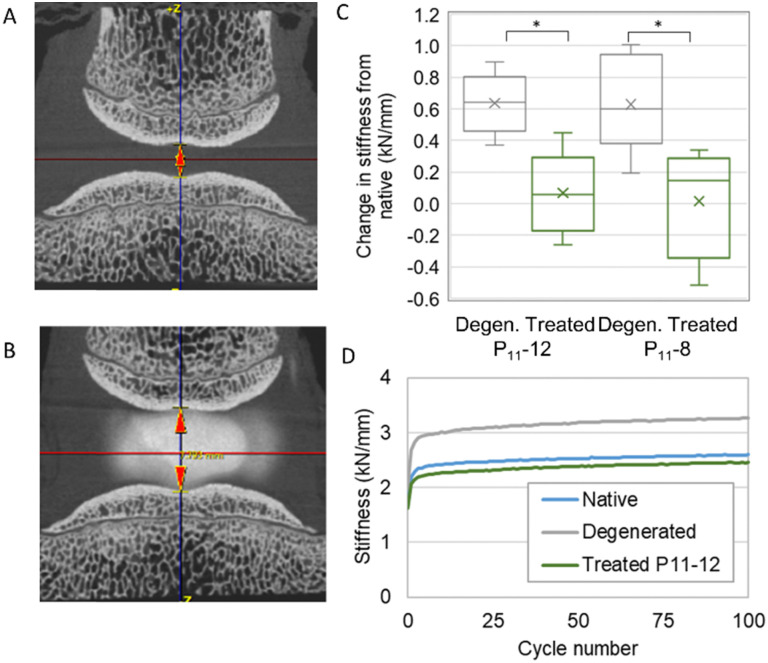
(A) and (B) MicroCT images of a specimen following artificial degeneration (D) and treatment (E), showing the increase in height following injection of the hydrogel. (C) Change in stiffness following degeneration and treatment. Specimens showed a significant increase in stiffness following artificial degeneration and a reduction to the native levels following treatment. (D) The stiffness behaviour over the 100 cycles in the three states for a typical specimen.

We developed a mechanical testing protocol that was sufficiently short in duration to enable specimens to be tested longitudinally in their native and degenerated states, as well as subsequently after nucleus augmentation (Fig. 6A and B). We found a significant reduction in specimen height between the native and artificially degenerated specimens (*n* = 12, *p* < 0.01) (Fig. S1, ESI†). Furthermore, we found significant differences in the specimen stiffness after testing for 100 axial loading cycles between the native and artificially-degenerated cases, providing two control values against which to compare the treated cases (*n* = 12, *p* < 0.01).

We went on to examine the mechanical performance of the two candidate peptide-GAG hydrogels in the degeneration model. In all cases, specimens were tested longitudinally in the native, artificially degenerated and peptide-augmented states. The hydrogels were injected manually using standard syringes with custom fixtures such that equal volumes of monomer and CS solution were delivered simultaneously through two 25 G 100 mm needles. A total volume of 0.3 mL was injected in all cases. The stiffness of the peptide-augmented specimens after 100 cycles was found to be not significantly different to the native specimens and significantly less stiff than the artificially degenerated specimens for both P_11_-12 and P_11_-8 (Fig. 6C), indicating that the peptides could restore biomechanical properties to the native levels. Furthermore, testing over longer periods revealed that while the stiffness of the artificially degenerated specimens reached a plateau after less than 8000 cycles (gradient < 0.01 N mm^−1^ per cycle), the stiffness of the native and augmented specimens continued to change to beyond 15 000 cycles (Fig. S2, ESI†). Examination of the disc height using micro computed tomography (microCT) showed that the drop in height caused by the degeneration step was restored following nucleus augmentation (Fig. 6A and B, Fig. S2, ESI†). No differences in mechanical performance or in height restoration were observed between the P_11_-8 and P_11_-12 hydrogels.

## Conclusion

Injectable biomaterials offer potential in the treatment of a number of soft tissue pathologies, but have to be designed to meet demanding requirements relating to both their deliverability and resulting properties. Previously, we have shown that a family of peptide-GAG hybrid hydrogels can be tuned to have appropriate mechanical properties for intervertebral disc nucleus repair. Here we extend the evidence to demonstrate the gels can be successfully delivered through a minimally invasive technique and self-assemble to form a gel *in situ*. Importantly, the resulting gel properties do not vary through either clinically relevant temperature range or needle size range. Furthermore, the injected hydrogel was shown to restore disc height and stiffness to native levels using a novel *in vitro* sequential testing regime under cyclic loading. This work provides *in vitro* evidence for the efficacy of the hydrogel system for nucleus augmentation prior to human tissue *in vitro* testing and *in vivo* studies.

## Author contributions

JPW, RHC, MPC and ARD performed the investigations and the formal analysis with MM. The work was conceptualised by RKW, DEM and PB and supervised by RKW, PB and MM. The manuscript was written, reviewed and edited through contributions from all authors.

## Data availability

Data for this article, including raw biomechanical and imaging data, processed data and images are available at Leeds Data Repository at https://doi.org/10.5518/1591.

## Conflicts of interest

There are no conflicts of interest to declare.

## Supplementary Material

MA-005-D4MA00613E-s001
