# Supplementary material for: Injectable peptide-glycosaminoglycan hydrogels for soft tissue repair: in vitro assessment for nucleus augmentation
Source: Mater Adv. 2024 Oct 10;5(21):8665–72. doi: 10.1039/d4ma00613e (PMC11474259; doi:10.1039/d4ma00613e)
Supplement: MA-005-D4MA00613E-s001 [file MA-005-D4MA00613E-s001.pdf]

Supplementary Information (SI) for Materials Advances.
This journal is © The Royal Society of Chemistry 2024

Electronic Supporting Information

Injectable peptide-glycosaminoglycan hydrogels for soft tissue repair: *in vitro* assessment for nucleus augmentation

James P. Warren, Ruth H. Coe, Matthew P. Culbert, Andrew R. Dixon, Danielle E. Miles, Marlène Mengoni, Paul A. Beales, Ruth K. Wilcox

Full Methods

Hydrogel Materials

The peptides were custom synthesised (CS Bio, USA). Peptide quality control was undertaken by the synthesis company. The peptide content reflects non-peptide molecules present in the dry peptide mass; these were mainly residual amounts of water and trifluoroacetic acid (TFA) counterions.

The glycosaminoglycan (GAG) used throughout this study was a chondroitin sulphate (CS) sodium salt from shark cartilage (Sigma Aldrich, UK).

Hydrogel Preparation Methods

To examine the effects of preparation, two methods were compared.

The established laboratory protocol[1] incorporated pH switching and heat monomerisation. The peptide powder and NaCl solution (130 mM) was vortexed and sonicated, followed by pH adjustment to 7.4 ± 0.05, heated to 80°C and further vortexed. The CS powder and NaCl solution (130 mM) was vortexed until dissolved. The two solutions were pipetted together and further vortexed until homogenous.

In the vortex only method, both the peptide-NaCl and CS-NaCl solutions were vortexed until clear and the peptide solution was then added to the GAG solution with further vortexing until homogenous.

For the needle delivery and rheology studies, the peptide and GAG solutions were separately prepared. Both peptide and GAG solutions were vortexed for 30 seconds, sonicated to remove air bubbles for 1-2 min, then drawn into 1 mL syringes and the relevant needle attached.

FTIR

Samples were placed between CaF_2 crystals and their spectra acquired four days after preparation with a Thermo Scientific Nicolet 6700 FTIR spectrometer. Spectra were averages of 32 scans recorded at room temperature. Blank solvent spectra were subtracted from the sample trace, the baseline corrected and the spectra smoothed. Processed spectra were band fitted in the amide I′ region (1720-1580 cm^{-1}) using the Peak Resolve routine in OMNIC7.3 SP1 (Thermo Electron Corporation), providing information on the number and positions of individual component bands. The peak positions corresponding to secondary structures used to determine β-sheet content are presented in Table S1.

Note that in the purification of peptides, trifluoracetic acid, TFA is used, which leads to it being present in the peptide material as a counter ion bound to the positively charged residues. TFA has a FTIR band located at 1673 cm^{-1} and peptides with greater number of arginine and ornithine residues will contain more TFA and therefore a large TFA peak in the FTIR spectra.

Table S1. Peak positions used to determine β-sheet content

Amide I′ band (cm^{-1})	Secondary structure assignment
1613-1630	β-sheet
1642-1649	Unordered
1649-1655	α- helix
1658-1674	Turn
1682-1690	Anti-parallel β-sheet
1694-1697	Turn

Rheology

Peptide-GAG samples were made by injecting the two individual solutions into a 2 mL Eppendorf using a syringe driver. Samples were made 24 hours prior to testing and maintained at room temperature before being loaded onto the rheometer using a custom increased diameter 1 mL Eppendorf pipette tip.

Rheology measurements used a Malvern Kinexus Pro rheometer with a cone-plate geometry (cone angle: 1°, diameter: 50 mm, gap: 0.03 mm). All tests were performed at 25 °C, utilizing a solvent trap. The atmosphere within was kept saturated to minimize evaporation of the peptide samples. The geometry was lowered into position and samples incubated for 15 min. To ensure measurements were made in the linear viscoelastic regime, amplitude sweeps were performed in a shear strain controlled mode from 0.01-100% at 1 Hz and 20 Hz. The dynamic moduli of the hydrogels were measured as a frequency function with the sweeps carried out between 1 and 20 Hz.

The shear moduli for the gels produced using different needle configurations was compared using a two-way ANOVA tests with Tukey post-hoc analysis ($p \leq 0.05$). Statistical analysis is carried out using Origin 2019 software (OriginLab Corporation, USA).

Needle delivery studies

A few drops of food colouring were added to the peptide (blue) and CS (yellow) solutions prior to vortexing. Needles were inserted at different measured orientations into an Eppendorf and the two solutions were injected simultaneously. Photographs of the resulting gel were taken to examine the consistency of mixing.

Further tests were undertaken using a bovine tail bone-disc-bone unit. First, a trans-endplate nucleotomy was undertaken, as described by Sikora et al [2], in which a 10 mm diameter central region of the nucleus was removed by drilling through the superior vertebra and endplate, avoiding damage to the inferior endplate. A camera was mounted above the specimen allowing visualisation of the nucleus void. The peptide-GAG hydrogel was then injected into the void through two parallel 25G needles using a syringe driver, and the process filmed to observe the gelation.

Biomechanical testing

Bovine disc preparation: Bovine tails were harvested from calves aged less than 30 months at a local abattoir and frozen (-80°C) prior to experimentation. The tails were cleaned and imaged under microCT as per Sikora et al [2], to identify the positioning of the sections to ensure consistent 15 mm lengths of bone were retained on either side of the disc. The bone-disc-bone units were then excised via transverse cuts through the vertebrae. Specimens were prepared by cleaning the blood and marrow using a water pik followed by a 24 hours soak in sodium citrate solution (20.5mM, pH 7.4) under agitation at 4°C. Prepared specimens were frozen until testing at -80°C.

Mechanical testing: The following steps were then undertaken on the specimens in their native state and again after artificial degeneration and after treatment. First, to allow the specimens to reach osmotic equilibrium prior to mechanical testing, they were held under a ~40 N load in a PBS bath at 37°C for 24 hours using a custom rig. Cyclic compression tests were then undertaken on a dynamic materials testing machine (ElectroPuls E10000, Instron, UK) under load control between 356 and 744 N at 1 Hz for 100 cycles. During testing, specimens were immersed in a PBS bath at 37 °C and porous fixtures were used to allow fluid flow through the endplates.

Degenerative model: The specimens were artificially degenerated by injecting a concentrated papain solution (0.3 ml, 1.6 kU/ml) to non-selectively break down collagen and proteoglycan structures within the nucleus pulposus. The papain solution was injected through a 30G needle into the nucleus of the disc and the specimens were then held under a ~40 N load at 42°C for 24 hours, followed by the injection of an ebselen inhibitor solution (0.3 ml, 2.13 mM) to stop further enzymatic activity.

Hydrogel injection: Peptide-GAG hydrogels were injected as a 2-part solution through two 25G needles aligned in parallel. Tests were undertaken on both P_{11}-8 and P_{11}-12 (both at 1:20 peptide:GAG ratio); a radio-opaque agent (Ultravist® 300, Bayer PLC, Reading, UK) was mixed with NaCl solution (130 mM) and carboxyfluorescein NaCl solution (2.7 mM) in a ratio of 1:2:1. This solution was then mixed with the hydrogel prior to injection to enable visualisation using microCT.

Imaging: Specimens were imaged during the testing sequence using micro-CT (uCT100, Scanco Medical, Switzerland).

Data analysis: Cyclic data was post-processed to extract specimen stiffness over the loading portion of each cycle, and the average stiffness over the last 10 cycles (i.e. at closest to steady-state) was used for analysis. The specimen height was also measured at each stage of testing. A two-factors repeated measure ANOVA was used to compare the stiffness values or the specimen height across the three testing stages and the two types of peptide used. Statistical analysis was performed using R.4.1.1 (R Project for Statistical Computing, R Foundation, www.r-project.org) after testing for data normality with a Shapiro-Wilk test and model sphericity with a Mauchly test.

Supplementary Results

Mechanical *in vitro* testing

The extended cycle testing out to 20,000 and 60,000 cycles for specimens under different conditions is shown in Figure S1. Note for specimens in the Degenerated and Treated states, the previous phases of the testing were undertaken to 1,000 cycles.

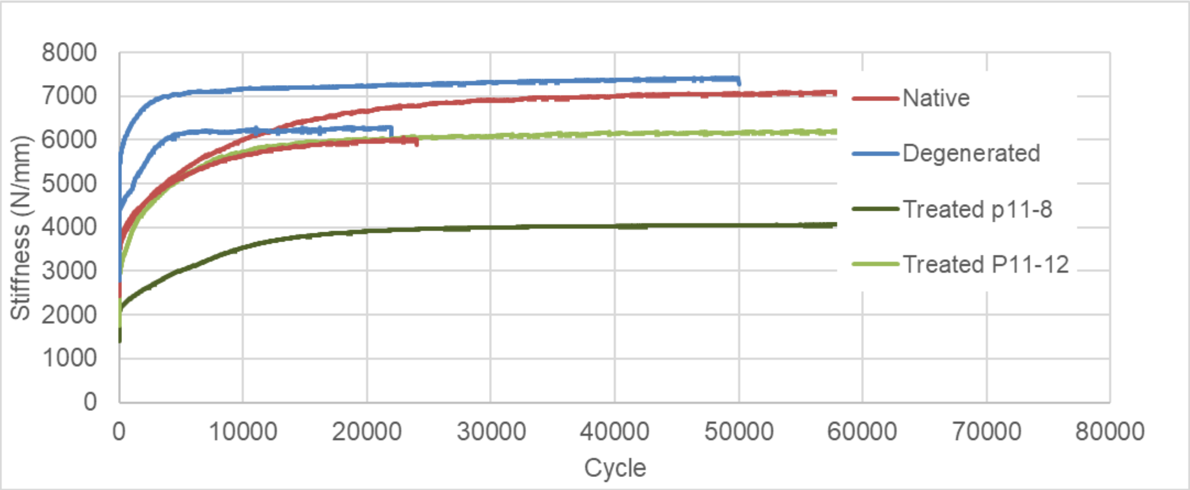

Figure S1. The stiffness of each load cycle of bone-disc-bone specimens tested in their native state or following artificial degenerated or treatment.

The change in specimen height following artificial degeneration and peptide augmentation is shown in Figure S2.

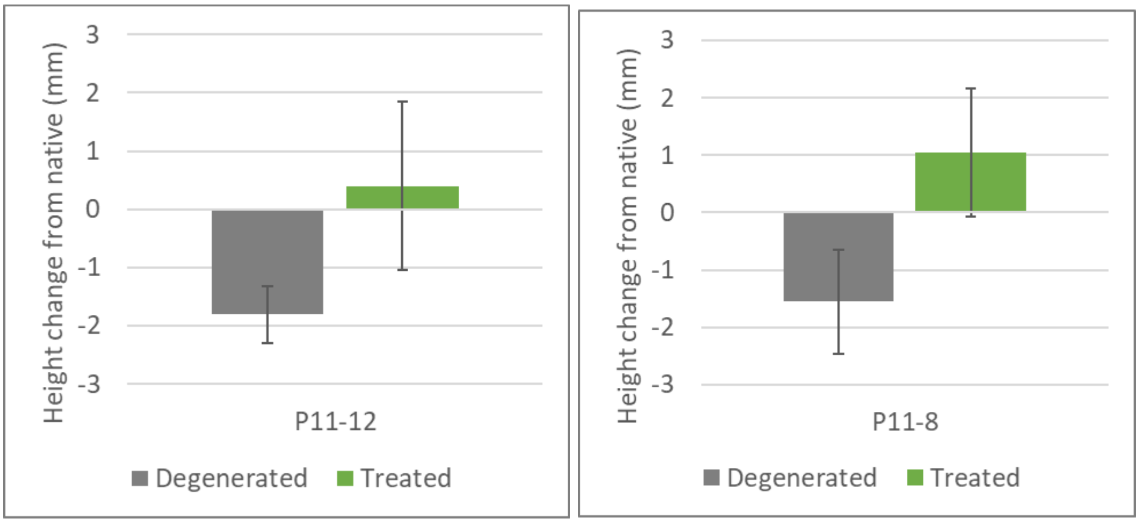

Figure S2 Change in the measured height of the specimen following artificial degeneration and following subsequent treatment with P_{11}-12 or P_{11}-8, following 100 cycles of load in each case; n = 6 for each hydrogel.

References

1. D. E. Miles, E. A. Mitchell, N. Kapur, P. A. Beales and R. K. Wilcox, J Mater Chem B, 2016, 4, 3225-3231.
2. S. N. Sikora, D. E. Miles, S. Tarsuslugil, M. Mengoni and R. K. Wilcox, Proc Inst Mech Eng H, 2018, 232, 230-240.
